# Top-down motivation both decreases and increases feature interference following a saccade

**DOI:** 10.3758/s13423-026-02907-6

**Published:** 2026-04-15

**Authors:** Tzu-Yao Chiu, Julie D. Golomb

**Affiliations:** Department of Psychology, The Ohio State University, 1835 Neil Avenue, Columbus, OH 43210 USA

**Keywords:** Motivation, Remapping, Dynamic attention, Feature interference

## Abstract

Spatial attention is rarely static, but instead highly dynamic in real-life. Recent studies have revealed that shifts of attention and eye movements incur consequences for ongoing feature perception, such as systematic feature-binding (swapping and mixing) errors. Here we investigated if these consequences are sensitive to top-down control, such that they could be flexibly mitigated when participants are motivated to improve their performance, or if feature interference reflects a more automatic byproduct of attentional remapping mechanisms following a saccade. In a preregistered experiment, 40 healthy adults reported the color of an item appearing at a pre-cued spatiotopic location. Following the cue, participants made a saccade, and then an array of colored items briefly appeared at the spatiotopic target location, retinotopic nontarget location, and two control nontarget locations. Crucially, across blocks we provided two different levels of performance-contingent reward (monetary incentives for precise spatiotopic target reports) to induce differences in top-down motivation. Compared to baseline lower-reward blocks, during higher performance-contingent reward (“bonus”) blocks, participants showed improved general performance and reduced subtle retinotopic mixing errors immediately after the saccade. However, we found the opposite effect on retinotopic swapping errors, with higher reward/motivation associated with increased retinotopic swapping errors early after the saccade, indicating that retinotopic swapping errors may reflect a structural limitation of the attentional remapping process. Overall, these results uncover effects of motivation on distinct attentional mechanisms underlying spatial remapping across saccades, and provide novel insights into the role of top-down control in dynamic spatial attention and visual perception more generally.

**Supplementary Information:**

The online version contains supplementary material available at 10.3758/s13423-026-02907-6.

Real-world perception and behavior are highly dynamic. For example, during natural viewing behavior, our eyes are rarely at rest but instead are moving constantly via ballistic eye movements, known as saccades, to sample visual information in the environment (Dorr et al., 2010). Humans sample the environment frequently, at a rate of multiple times per second, and also optimally, selecting targets according to natural scene statistics (Najemnik & Geisler, 2005). However, despite the apparent benefits afforded by making saccades, the resulting shifts in retinal inputs received across saccades pose a fundamental challenge to our visual system: How do we maintain stable visual perception whilst making saccades to sample the external world?

To date, extensive research has been conducted to understand perceptual mechanisms that aid in the maintenance of stability across saccades (Binda & Morrone, 2018), yet successful navigation in everyday life involves not only the intake of perceptual information but also the ability to exert top-down attentional control according to current behavioral goals (Braver, 2012; Posner, 1980). Maintaining a stable attentional focus across saccades is particularly challenging because after each saccade, spatial attention needs to be remapped/updated from the previously attended retinotopic (eye-centered) location to reflect the spatiotopic (world-centered) location (Golomb & Mazer, 2021).

Previous studies on transsaccadic attentional remapping have shown that attention remaps imperfectly across saccades (Golomb et al., 2008, 2010; Harrison et al., 2024; but see Rolfs et al., 2011). In a behavioral study, Golomb et al. (2008) asked participants to maintain attention at a cued spatiotopic location across a saccade and measured attention at different locations and timepoints after the saccade. Early after saccade completion, attentional facilitation lingered at the cue’s (now task-irrelevant) retinotopic location, which gradually decayed as postsaccadic delay increased. More recently, studies further investigated the perceptual consequences of this retinotopic attentional trace by implementing the continuous feature report paradigm (Wilken & Ma, 2004; Zhang & Luck, 2008). Golomb et al. (2014) cued participants at a peripheral location before a saccade and presented an array of colored items at different timepoints after the saccade. When asked to report the target at the cue’s spatiotopic location, two distinct types of systematic feature-binding errors were found early after saccade. First, participant’s target reports were found to be subtly biased towards the color of the nontarget item at the cue’s retinotopic location (mixing error). In addition, participants were also more likely to make large misreport errors of the retinotopic nontarget color compared with a control nontarget color (swapping error). These retinotopic feature-binding errors, early after saccade completion, suggest that task-irrelevant features at the previously attended retinotopic location interfere with ongoing perception of the goal-relevant object early after saccades (see also Chiu et al., 2025; Dowd & Golomb, 2020; Golomb, 2019). Furthermore, the retinotopic feature distortion errors (i.e., subtle mixing errors) were also observed when participants split covert attention, whereas the retinotopic swapping errors were observed when participants shifted covert attention. Together, the retinotopic feature-binding errors were thus interpreted to reflect distinct aspects of attentional remapping after saccades. More broadly, the postsaccadic time period represents one of multiple instances in which attention in dynamic contexts interferes with ongoing perception (e.g., attentional capture; Chen et al., 2019).

The presence of retinotopic feature interference following saccades, together with findings of persistent retinotopic effects in perception (Knapen et al., 2009; Mathôt & Theeuwes, 2013), attention (Golomb et al., 2008), and object representation (Gardner et al., 2008; Golomb & Kanwisher, 2012), suggests that the human visual system operates predominantly in eye-centered coordinates. This retinotopic dominant design of the visual system and its inefficiency in remapping to spatiotopic coordinates (Golomb et al., 2008) seem surprisingly suboptimal for maintaining stability across saccades, and in contrast with our routine experience of stable spatial representations in real life.

One of the most critical aspects of human cognition is cognitive control—the ability to regulate thoughts and actions in accordance to internal goals (see Braver, 2012, for review)—yet the influences of top-down motivational factors has rarely been tested in the context of transsaccadic visual stability. Previous studies have shown that various aspects of cognition, such as response preparation (Adkins & Lee, 2021, 2024) and working memory maintenance (Jimura et al., 2010; Wallis et al., 2015), can be enhanced when participants are motivated with high task incentives. Furthermore, it has been shown that monetary reward modulates spatial attention in the absence of eye movements, such that the prospect of performance-contingent reward biases attentional processes (Engelmann et al., 2009; Engelmann & Pessoa, 2007; Esterman et al., 2014, 2016; Leber & Irons, 2019; Shomstein & Johnson, 2013; van den Berg et al., 2014).

Might the retinotopic feature interference following saccades be similarly malleable to top-down reward/motivation, offering a clue into visual stability? We adopted Golomb et al.’s (2014) feature report paradigm to measure postsaccadic feature perception and implemented a performance-contingent reward scheme to manipulate participants’ motivation towards maintaining a precise spatiotopic representation. If feature-binding errors associated with the retinotopic trace are simply a form of diminished attentional performance over which the visual system can exert active control, the prevalence of feature-binding errors should be reduced (or even eliminated) under higher motivation. This default hypothesis predicts that increasing performance-contingent reward should decrease errors in the task, including general performance lapses (e.g., random guesses) as well as systematic feature-binding errors shortly after saccades. Conversely, it is possible that the retinotopic feature-binding errors are a more automatic byproduct of the postsaccadic remapping process, and might be more immune to the effects of top-down reward/motivation. Given that the retinotopic attentional trace has been suggested to arise from passive decay of residual neural activity at the previously attended location (Khayat et al., 2006; Wang, 2001), increased performance-contingent reward might even strengthen the allocation of attention before the saccade, resulting in a stronger or more persistent retinotopic trace, which could counterproductively produce an increase in retinotopic mixing and/or swapping errors.

## Methods

### Participants

The preregistered sample size was 40, determined based on power analysis performed on data from Golomb et al. (2014) without reward manipulations. The power analysis was performed using the *stats* package in R environment (Version 4.1.2; R Core Team, 2021). The mixing errors were characterized by the mean shift parameter ($\mu$) significantly above zero (one-sample *t* test), and the swapping errors were characterized by a significantly higher swap rate for the retinotopic color (${\beta }_{1}$) than that for the control nontarget color (${\beta }_{2}$) (paired-sample *t* test). The effects of interest had effect sizes of *d* = 0.74 and 0.59, respectively. Results of the power analysis suggested that 33 participants were required to detect both effects with 0.9 power. We set sample size at 40 to be conservative. A total of 45 students completed the experiment. Five participants’ data were excluded based on the exclusion criteria (see *Data Analysis*), resulting in 40 participants’ data analyzed (age: *M* = 20, *SD* = 2; gender: 12 men and 28 women).

### Apparatus

Stimuli were displayed using a 24-in. LCD monitor (240 Hz refresh rate). Screen resolution was 1,024 × 768 pixels. Viewing distance was 63 cm, at which approximately 28 cm occupied 1° visual angle. Participants’ eye gaze position from one eye was recorded using an EyeLink 1000 eye-tracker (1000 Hz sampling rate). We used chinrest and forehead rest to minimize participants’ head movement. The experiment and eye-tracker were controlled using MATLAB, Psychtoolbox (Brainard, 1997), and EyeLink Toolbox (Cornelissen et al., 2002).

### Stimuli

Stimuli included a fixation dot (0.48° diameter), a spatial memory cue (2° × 2° black square) indicating the target item’s location, the stimulus array (four 2° × 2° filled color squares), masks (four 2° × 2° squares with random color pixels), a color wheel in CIEL*a*b* space (L* = 60, a* = 22, b* =  − 1, radius = 50; 8.2° diameter), and a white feedback bar (2° length). The fixation dot appeared at four possible locations, positioned at corners of an invisible 10.5° × 10.5° rectangle centered on the screen, to guide participants’ eye movements. The saccades were constrained to be 10.5° in amplitude (i.e., horizontal or vertical saccades). There were nine possible stimulus locations, spaced evenly around the fixation positions. The stimulus array always appeared at the four stimulus locations surrounding the current fixation dot (7.4° eccentricity). The memory cue appeared at one of five possible spatiotopic stimulus locations (center, center-top, center-bottom, left-center, right-center), such that a stimulus could always appear at the cue’s spatiotopic and retinotopic locations after a saccade.

### Procedure

The experiment involved two trial types—saccade and no-saccade trials—randomly intermixed throughout the experiment. The experiment was organized into 12 blocks of 60 trials (40 saccade trials and 20 no-saccade trials). Before the experiment, the eye-tracker was calibrated using the 9-point grid method. Trials in which participants failed to follow the fixation dot (i.e., eye-position > 2° away from fixation dot or saccade latency > 500 ms) were stopped and repeated later in the same block.

#### Trial procedure

**Fig. 1:**
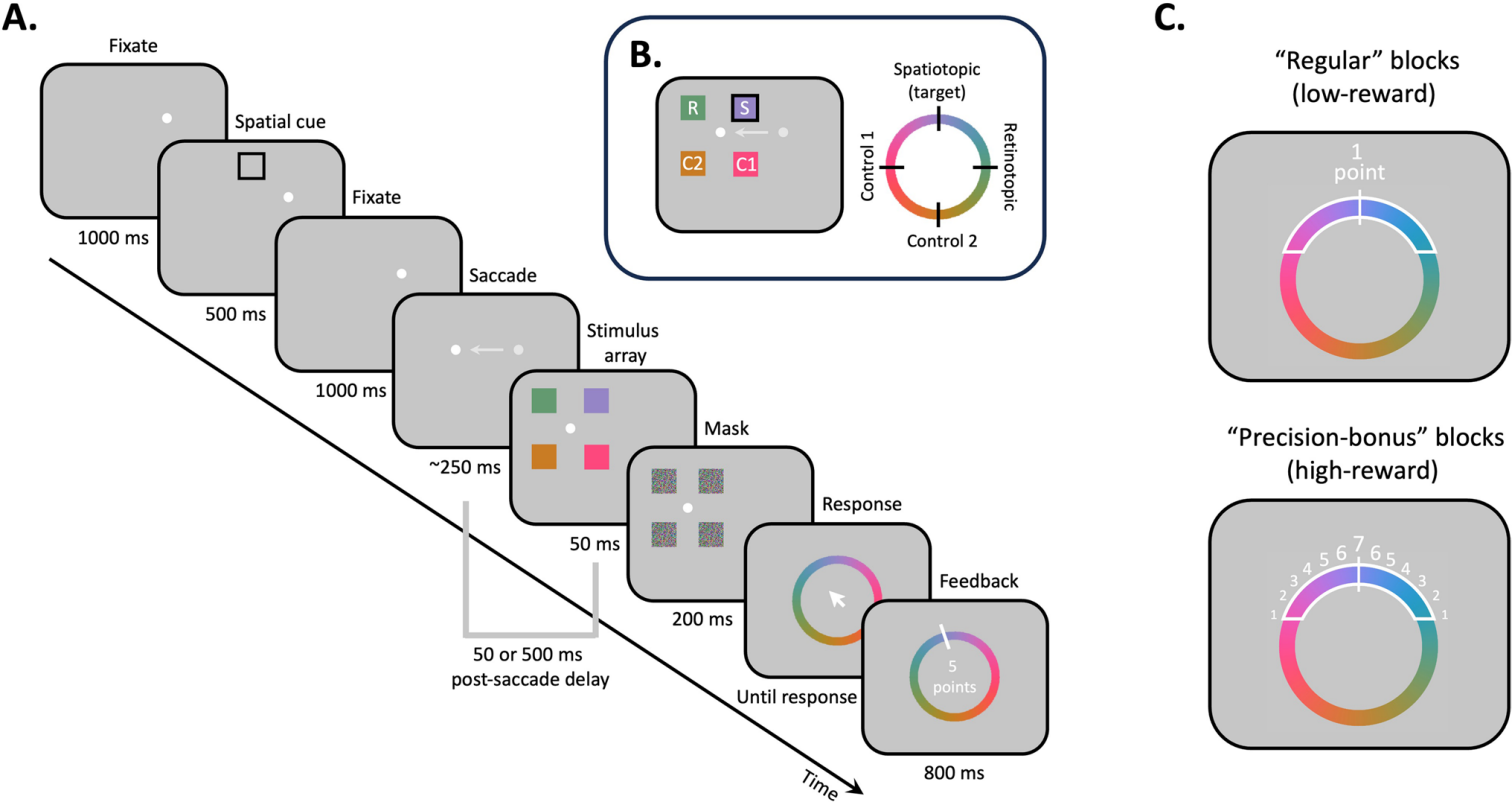
A. Procedure illustrating a sample saccade trial (see text for details). Note that trials proceeded in a gaze-contingent manner. **B.** Condition labels and color selections for the stimulus array in the example trial. One of the four color squares appears at the spatial cue’s spatiotopic location (the target item), one appears at the cue’s retinotopic location, one appears at an equidistant control location (control nontarget 1), and one appears diagonal away from the spatial cue’s spatiotopic location (control nontarget 2). The spatiotopic color is randomly selected on each trial. The retinotopic and control nontarget 1 color are selected to be 90° away from the spatiotopic color, whereas the control nontarget 2 color is 180° away from the spatiotopic color. S = Spatiotopic; R = Retinotopic; C1 = Control nontarget 1; C2 = Control nontarget 2. **C.** Reward manipulation. Low-reward blocks were described to participants as “regular” blocks, whereas high-reward blocks were described as “precision-bonus” blocks. Note that the rewarded zone is for illustration purpose and not scaled to the actual rewarded zone (30°) in the experiment. (Color figure online)

Figure 1A depicts an example of the saccade trial, which proceeded in a gaze contingent manner. Trials commenced when participants fixated on the fixation dot. After maintaining fixation for 1,000 ms, the memory cue appeared for 500 ms. Participants were told to covertly attend to the cued location and prepare to report the color of the item that subsequently appeared at this location. After the memory cue disappeared, participants had to keep fixating for another 1,000 ms. On the saccade trials (two-thirds of the trials), the fixation dot then jumped to a second location, serving as the saccade cue. Participants were instructed to follow the fixation dot as soon as possible and were allowed 500 ms to complete the saccade. Upon saccade completion, the memory array (four color squares with different colors) appeared at equidistant locations (7.4° eccentricity) around the fixation dot. The entire memory array appeared simultaneously, and onset at either an early (50-ms delay) or late (500-ms delay) postsaccadic timepoint. One of the memory items appeared at the spatiotopic, world-centered location of the memory cue—participants were asked to report the color of this item (target). Another memory item appeared at the same retinotopic (eye-centered) location of the memory cue, whereas the two remaining items appeared at mirror-symmetric control locations (see Fig. 1B).

On each trial, the color of the spatiotopic target item was randomly selected from 180 colors evenly distributed along the color wheel. Colors of the remaining items were chosen to make the retinotopic and equidistant control locations equally different from the spatiotopic color, but rotated in opposite directions (90° clockwise or counter-clockwise, randomly determined on each trial). The diagonal control color was always 180° away in color space. The memory array was shown for 50 ms, after which masks appeared for 200 ms at the same locations. After the masks disappeared, a color wheel (randomly rotated) and the cursor appeared at screen center. Participants were asked to report the target color by clicking on the color wheel and were given unlimited time to respond. After the response, a white line indicating the correct target color and the rewarded points (see *Reward Manipulation*) were shown. The intertrial interval was 1,300–1,700 ms.

Intermixed with the saccade trials were the no-saccade trials (one-third of the trials). The trial procedure was similar to the saccade trials except the fixation dot remained at its initial location. Instead of the saccade cue, an additional 350-ms fixed delay was included to mimic the saccade timing, followed by a jittered delay between 50 and 500 ms to match postsaccade timing. Duration of the jittered delay was randomly sampled and treated as a single timing condition, since performance was found to be similar across various delay timepoints on no-saccade trials in this paradigm (Golomb et al., 2014). One of the memory items appeared at the location of the memory cue (spatiotopic and retinotopic locations are identical since no saccade was made). The remaining three items appeared at two equidistant control locations and the diagonal control location.

#### Performance-contingent reward manipulation

We implemented a performance-contingent reward scheme to manipulate participants’ motivation and incentivize accurately reporting the color at the spatiotopic target location. Participants accumulated points throughout the experiment that were transformed into a monetary bonus after the experiment. The performance-contingent reward manipulation included two conditions (low reward and high reward) and was blocked (Fig. 1C). Participants were informed before each block whether the upcoming block was a “regular” block (low-reward) or a “precision-bonus” block (high reward). In “regular” (low reward) blocks, participants received one point for each response that was reasonably close to the spatiotopic target color (error < 30°), and zero points for responses outside of this range. In “precision-bonus” (high reward) blocks, participants received additional points for more precise responses. Here they received between one to seven points for each response reasonably close to the target color (error < 30°), with the number of points increasing linearly as error decreases (maximum 7 points for errors < 4°). The goal was to increase participants’ motivational state (i.e., a “try harder” signal) with higher reward prospect on the high-reward blocks (Engelmann et al., 2009; Engelmann & Pessoa, 2007; Esterman et al., 2014, 2016; Leber & Irons, 2019; van den Berg et al., 2014). Participants were shown after each response how many points they earned on that trial. After each block, participants were told how many dollars they had according to the accumulated points. Participants performed six low-reward blocks and six high-reward blocks in random order.^1^

### Design

The experiment had a 2 (low-reward, high-reward) × 3 (no-saccade, saccade early delay, saccade late delay) within-participant design. All six conditions were balanced across the experiment, with 120 trials assigned to each condition.

### Data analysis

#### Data transformation and model fitting

Response error was calculated as the difference between the reported and correct target color values; realigned directionally on saccade trials such that the spatiotopic target color was represented at 0°, the retinotopic nontarget color at + 90°, and the equidistant control nontarget color at − 90°. For no-saccade trials, the two equidistant control colors were represented at + 90° and − 90° (randomly assigned). The distribution of response errors from each participant in each condition was fitted to probabilistic mixture models (Bays et al., 2009; Zhang & Luck, 2008) using the MemToolbox (Suchow et al., 2013). Kolmogorov–Smirnov tests were then run on each individual model fit to ensure good fits to the raw data (all *p* values > .06). As preregistered, we fitted the saccade trial data to the model described below, which has been previously shown to capture both sources of retinotopic feature interference (i.e., mixing and swapping errors; Golomb et al., 2014). Note that although there is recent evidence questioning the use of mixture models to draw theoretical distinctions between response precision measured by the standard deviation parameter of the primary target distribution and random guessing measured by the uniform distribution parameter (Schurgin et al., 2020), our main analyses do not focus on these parameters or this theoretical interpretation. Rather, we focus on directional differences in swap and shift parameters associated with systematic feature-binding errors (Chen et al., 2019; Golomb et al., 2014; Narhi-Martinez et al., 2023). That said, we also performed exploratory analyses on unmodeled measures (Supplementary Analyses).

$$p\left(\theta \right)=\left(1-{\beta }_{1}-{\beta }_{2}-\gamma \right){\phi }_{\mu , \kappa }+{\beta }_{1}{\phi }_{+90^\circ , \kappa }+{\beta }_{2}{\phi }_{-90^\circ , \kappa }+\gamma \left(\frac{1}{360^\circ }\right)$$

The formula above defines the saccade trials’ mixture model including systematic feature-binding errors (Golomb et al., 2014), with three von Mises (circular Gaussian) distributions $(\phi )$, centered on (1) the spatiotopic target color (with flexible mean $\mu$ around zero, constrained between − 30° and + 30°), (2) the retinotopic nontarget color (mean fixed at + 90°), and (3) the equidistant control nontarget color (mean fixed at − 90°), plus a uniform guessing distribution. ${\beta }_{1}$ reflects the probability of misreporting the retinotopic nontarget color, ${\beta }_{2}$ reflects the probability of misreporting the equidistant control nontarget color, and $\gamma$ reflects the probability of random guessing. The three von Mises distributions are set to have the same concentration κ (*SD* = $\sqrt{1/\upkappa }$). Data from the no-saccade trials were fitted to a basic mixture model (guess rate and standard deviation).

#### Exclusion criteria

As preregistered, we excluded participants based on their performance on no-saccade trials (pooled across levels of reward). Participants were excluded if their probability of randomly guessing ($\gamma$) exceeded 0.5 or their standard deviation exceeded 80° (preregistered criteria) since meeting these criteria indicated that the participant was unable to perform the task sufficiently (e.g., making random guesses on more than 50% of the trials). These criteria are also consistent with previous studies adopting similar paradigms (Golomb et al., 2014; Narhi-Martinez et al., 2023, 2024).

## Results

**Fig. 2:**
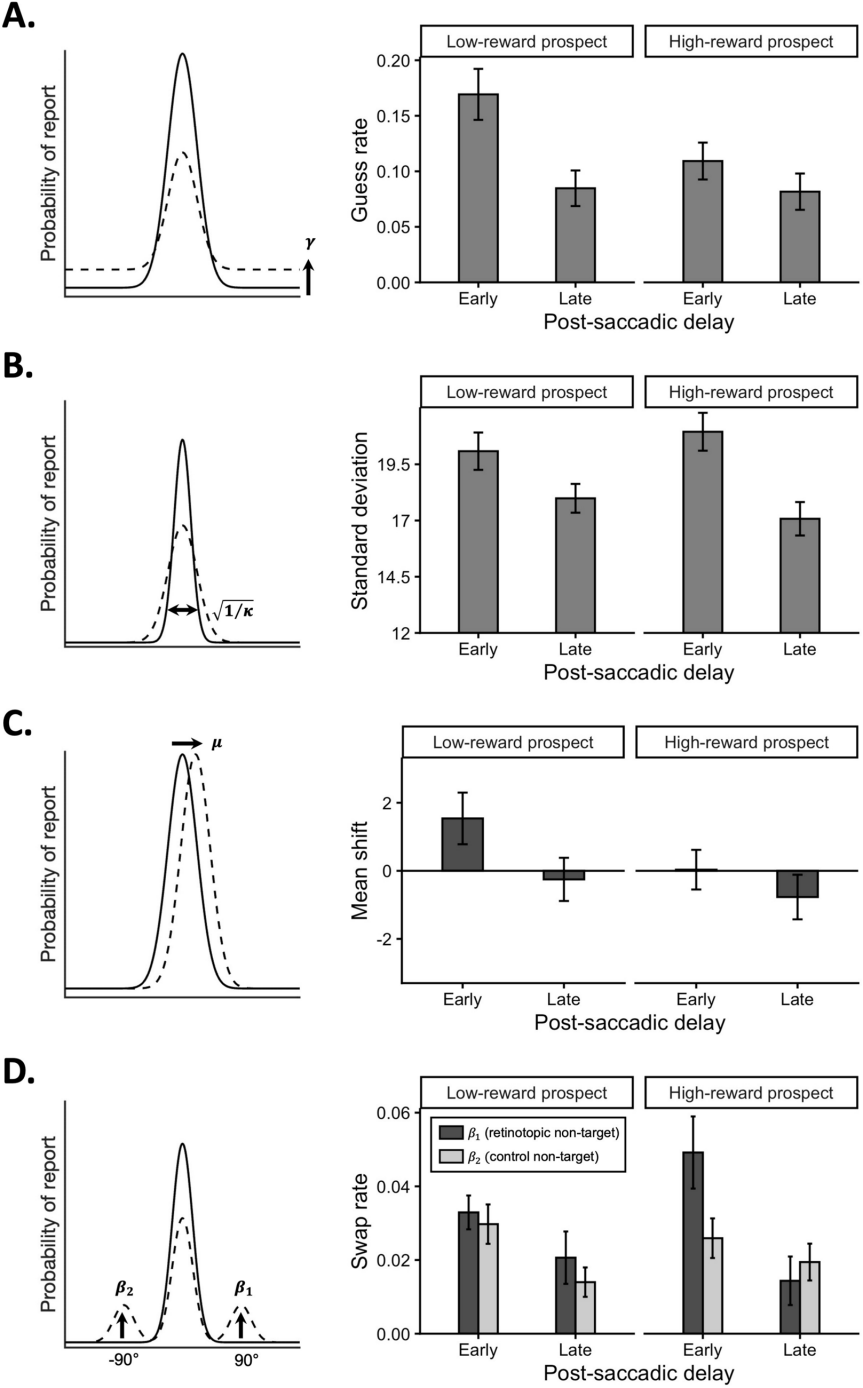
Cartoon illustrations for each parameter in the model and mean maximum likelihood parameter estimates for each condition. Low-reward prospect corresponds to the “regular” blocks, whereas high-reward prospect corresponds to the “precision bonus” blocks. **A.** Illustration and results on guess rate ($\gamma$). **B.** Illustration and results on standard deviation ($\sqrt{1/\kappa }$). **C.** Illustration and results on mean shift ($\mu$). **D.** Illustration and results on swap rates ($\beta$). ${\beta }_{1}$ reflects the probability of misreporting the retinotopic color, ${\beta }_{2}$ reflects the probability of misreporting the control nontarget 1 color. Error bars represent 95% confidence interval calculated based on within-participant standard error of the mean (Cousineau, 2005)

Mean maximum likelihood parameter estimates for each model parameter and condition are depicted in Fig. 2. We start by testing the effects of reward prospect and postsaccadic delay on general performance indicators (Fig. 2A–B), then turn to the critical examinations of systematic feature-binding errors (Fig. 2C–D).

Firstly, to examine whether reward prospect and postsaccadic delay influenced general performance, we performed two-way repeated-measure analyses of variance (ANOVAs) on guess rate ($\gamma$; Fig. 2A) and standard deviation ($\sqrt{1/\kappa }$; Fig. 2B). Consistent with prior studies, general performance was impaired when the stimulus array was presented immediately following a saccade compared with at a longer postsaccade delay, with significant main effects of postsaccadic delay for guess rate, *F*(1, 39) = 23.1, *p* < .001, ${\eta }_{p}^{2}$ = 0.37, *BF*_*incl*_ = 442.86, and standard deviation, *F*(1, 39) = 32.96, *p* < .001, ${\eta }_{p}^{2}$ = 0.46, *BF*_*incl*_ = 13,259.34. Results on guess rate showed a significant main effect of reward prospect, *F*(1, 39) = 10.69, *p* = .002, ${\eta }_{p}^{2}$ = 0.22, *BF*_*incl*_ = 4, with participants less likely to make random guesses when reward prospect was higher, and a significant interaction, *F*(1, 39) = 6.98, *p* = .012, ${\eta }_{p}^{2}$ = 0.15, *BF*_*incl*_ = 12.55. Post hoc tests showed a significant reduction in guess rate with higher reward prospect at the early postsaccadic timepoint, *t*(39) = 3.58, *p*_*bonf*_ = .002, *d* = 0.57, *BF*_10_ = 31.96, but not at the late timepoint, *t*(39) = 0.26, *p*_*bonf*_ = 1, *d* = 0.04, *BF*_10_ = 0.18. Results on standard deviation showed no main effect of reward prospect (*F* < 0.01, *p* = .958, ${\eta }_{p}^{2}$ < 0.01, *BF*_*incl*_ = 0.21), but a significant two-way interaction, *F*(1, 39) = 5.03, *p* = .031, ${\eta }_{p}^{2}$ = 0.11, although Bayesian statistics suggested only anecdotal evidence (*BF*_*incl*_ = 2.97). Post hoc tests showed that the effect of reward prospect on standard deviation was not reliable at either delay timepoint (*p*s_bonf_ > .1, *BF*s_10_ < 1), whereas the effect of postsaccadic delay was significant in both reward conditions (*p*s_bonf_ < .015, *BF*s_10_ > 19), suggesting the weak interaction effect might be driven by a slightly larger delay effect in the high-reward condition than the low-reward condition.

More importantly, in order to investigate systematic feature errors (i.e., retinotopic mixing and swapping errors; Fig. 2C–D) and whether these errors are influenced by reward prospect and postsaccadic delay, we performed one-sample *t* tests and two-way repeated-measure ANOVAs on mean shift ($\mu$) and retinotopic swapping errors (${\beta }_{1}-{\beta }_{2}$). In the low-reward condition, we found significant mean shift towards the retinotopic color in the early delay condition, *t*(39) = 2.81, *p* = .008, *d* = 0.44, *BF*_10_ = 5.09, and no significant mean shift in the late delay condition, *t*(39) =  − 0.73, *p* = .467, *d* =  − 0.12, *BF*_10_ = 0.22, replicating previous findings in the absence of performance-contingent reward suggesting the presence of mixing errors temporally limited to the early postsaccadic timepoint. Critically, in the high-reward condition, we found no significant mean shift in the early delay condition, *t*(39) = 0.08, *p* = .938, *d* = 0.01, *BF*_10_ = 0.17, and a significant mean shift in the opposite direction, away from the retinotopic color, in the late delay condition, *t*(39) =  − 2.88, *p* = .006, *d* =  − 0.46, *BF*_10_ = 6. The overall shift away from the retinotopic color in the high-reward condition compared with the low-reward condition was supported by a significant main effect of reward prospect, *F*(1, 39) = 9.76, *p* = .003, ${\eta }_{p}^{2}$ = 0.2, *BF*_*incl*_ = 7.11. The main effect of postsaccadic delay was significant, *F*(1, 39) = 6.21, *p* = .017, ${\eta }_{p}^{2}$ = 0.14, *B*_*Fincl*_ = 3.39, indicating a stronger mean shift towards the retinotopic color in the early delay condition than in the late delay condition. The two-way interaction was not significant, *F*(1, 39) = 3.1, *p* = .086, ${\eta }_{p}^{2}$ = 0.07, *BF*_*incl*_ = 1.

For the swapping errors, we found no significant retinotopic swapping (over and above the baseline control swap rate) at either delay timepoint in the low-reward condition—Early: *t*(39) = 0.56, *p* = .577, *d* = 0.09, *BF*_10_ = 0.2; Late: *t*(39) = 1.35, *p* = .185, *d* = 0.21, *BF*_10_ = 0.39. In the high-reward condition, we found significant retinotopic swapping at the early delay timepoint only—Early: *t*(39) = 3.75, *p* = .001, *d* = 0.59, *BF*_10_ = 50.31; Late: *t*(39) =  − 1.21, *p* = .232, *d* =  − 0.19, *BF*_10_ = 0.34, indicating that when performance-contingent reward prospect was relatively high, participants were more likely to exhibit the previously reported pattern of misreporting the retinotopic color compared with the control nontarget color early after saccade. The Reward × Delay interaction was significant, *F*(1, 39) = 10.26, *p* = .003, ${\eta }_{p}^{2}$ = 0.21, *BF*_*incl*_ = 176.35, and post hoc tests showed that the effect of reward prospect was limited to the early delay condition. Unlike the other types of errors, retinotopic swapping errors in the early delay condition were significantly *more likely* under high compared to low reward prospect, *t*(39) = −2.86, *p*_*bonf*_ = .014, *d* =  − 0.45, *BF*_10_ = 5.66. No significant difference was found in the late delay condition, *t*(39) = 2.15, *p*_*bonf*_ = .076, *d* = 0.34, *BF*_10_ = 1.33. The main effect of postsaccadic delay was significant, *F*(1, 39) = 7.07, *p* = .011, ${\eta }_{p}^{2}$ = 0.15, albeit Bayesian statistics suggesting inconclusive evidence (*BF*_*incl*_ = 2.23), and the main effect of reward prospect was not significant, *F*(1, 39) = 1.17, *p* = .287, ${\eta }_{p}^{2}$ = 0.03, *BF*_*incl*_ = 0.27.

In short, we found that incentivizing performance with higher performance-contingent reward for the spatiotopic task prevents the occurrence of subtle mixing errors but counterproductively increases retinotopic swapping errors shortly after saccade completion. In addition, we performed exploratory analyses to examine the possibility of parameter tradeoff in model fitting. We found no reliable relationship between the parameters reflecting retinotopic feature interference (i.e., $\mu$ vs. ${\beta }_{1}$) and similar patterns of retinotopic swapping errors with an alternative model without the flexible mean shift ($\mu )$ parameter, together indicating that parameter tradeoff cannot explain the observed differences across reward conditions on postsaccadic feature-binding errors (Supplementary Analyses).

## Discussion

In this study, we integrated an incentive manipulation with continuous feature report to investigate how top-down motivation influences visual feature perception following saccades. Firstly, we found that increased reward prospect enhanced participants’ general performance on the task (e.g., reduced guess rate in higher performance-contingent reward blocks), consistent with prior research on motivation and the allocation of spatial attention without eye movements (Engelmann & Pessoa, 2007; Engelmann et al., 2009). Our more novel findings concern the effects of reward/motivation on the two distinct patterns of postsaccadic feature-binding errors: retinotopic swapping and retinotopic mixing errors. At the critical early postsaccadic timepoint where these errors typically emerge, participants showed reduced retinotopic mixing errors, but increased retinotopic swapping errors, in the high-reward condition relative to the low-reward condition. Previous research has found mixing and swapping errors in distinct contexts where participants split or shifted covert attention, respectively, leading these studies to suggest that they reflect separate attentional mechanisms that are both involved in postsaccadic remapping (Dowd & Golomb, 2019, 2020; Golomb et al., 2014). The intriguing patterns of feature-binding errors observed in the current study further suggest that top-down motivation can influence multiple components of attentional remapping following saccadic eye movements.

In terms of the mixing errors, the low-reward condition showed reliable mixing errors in the early postsaccadic delay condition but not in the late delay condition, consistent with findings from previous studies implementing a similar paradigm without reward manipulations (Golomb et al., 2014). In the high-reward condition, we observed a lack of mixing errors at the early postsaccadic timepoint, and somewhat unexpectedly, the presence of an opposite-direction repulsion bias away from the retinotopic color at the late postsaccadic timepoint. These findings suggest that postsaccadic feature mixing errors are malleable to active cognitive control. When motivated by higher incentive to maintain a precise spatiotopic representation across a saccade, the visual system might be able to prevent mixing of the competing retinotopic color early after saccade and potentially even suppress the retinotopic color at a later postsaccadic timepoint. Interestingly, similar subtle repulsion biases have been found in other contexts where participants may be explicitly trying to suppress or minimize competition between items—for example, studies on attentional capture, where reports of a target color can be biased away from the color of a salient distractor competing for spatial attention (Chen et al., 2019); studies on covert divided attention, where two similar colors may be encoded as slightly more distinct than they actually are (Bae & Luck, 2017; Golomb, 2015); and studies on working memory for multiple items, where repulsion bias increases over time during active maintenance (Scotti et al., 2021).

Conversely, results on swapping errors showed a detrimental reward/motivation effect. Participants made more retinotopic swapping errors when motivated by higher reward prospect for precise spatiotopic responses. This effect is unlikely to reflect decision-level strategies employed in the high-reward condition, since retinotopic swaps (or any nontarget misreport errors) were far outside of the rewarded color range and therefore would always result in zero points received on a given trial. Instead, the results could reflect the underlying attentional mechanisms of remapping. As noted earlier, retinotopic swapping errors are interpreted to occur when attention is still stuck at the previously attended retinotopic location, and more generally are found in contexts where the stimulus array is presented at a brief delay after a shift (or saccade) cue, such that on some trials the locus of attention has not had time to update to the new location. Based on this framework, one interpretation is that top-down motivation influences the strength of the initial, *pre-saccadic* attentional prioritization, perhaps consistent with a recent finding that monetary incentive affects resource allocation when proactive control can be engaged before encoding (Brissenden et al., 2023). In this case, higher reward prospect could incentivize stronger prioritization of the cued location *prior* to the saccade. Due to the recurrent nature of attention (Wang, 2001), this increase could in turn result in stronger residual activity at the original retinotopic location after the saccade that takes longer to decay, hence counterproductively inducing more retinotopic swapping errors early after saccade completion.

However, we note that an interpretation that only accounts for an effect of top-down motivation on presaccadic attention would not be a complete account of our findings. The fact that we found increases in retinotopic swap errors but decreases in retinotopic mixing errors suggests a more complex account, consistent with remapping involving multiple aspects/processes that might be differently influenced by top-down control. To elaborate, in order to maintain spatiotopic attention across saccades, the visual system needs to both allocate attention to the cued location before the saccade, and also remap the attentional focus after the saccade (which could itself involve both a rapid turning-on of attention at the new spatiotopic location and a slower turning-off at the old retinotopic location; Golomb, 2019). Such an interpretation is consistent with the neuronal dynamics of shifting covert attention in the absence of eye movements (Khayat et al., 2006), where attentional facilitation may “turn-on” at the new location before neural activity at the old location gradually “turns off.” If swap errors are interpreted as trials on which attention was still stuck at the previous retinotopic location and mixing errors are interpreted as trials on which attention may be split between retinotopic and spatiotopic locations (Golomb et al., 2014), then some possible explanations for our results include the higher reward condition potentially reflecting a change in the balance between the allocation of attention at the retinotopic trace and emerging spatiotopic locations (e.g., an either-or rather than splitting of attention); a change in the time course of attentional remapping (i.e., it is possible that top-down motivation might shift the postsaccadic time windows when these errors are found); or simply that on trials where spatial attention was temporarily split between the spatiotopic and retinotopic locations, participants may have been able to exert more control to suppress the task-irrelevant color (whereas the retinotopic swapping errors are a more automatic by-product of increased presaccadic attention). Interestingly, the results of early retinotopic swapping errors and late repulsion biases together may also be consistent with findings reported in the visual attention/search literature, where a distracting object was initially prioritized and subsequently inhibited (Cunningham & Egeth, 2016; Moher & Egeth, 2012; M. Posner & Cohen, 1984).

Taken together, these findings provide novel insights into the role of top-down cognitive control in transsaccadic perception. Future investigations might further unveil whether other critical transsaccadic processes—including transsaccadic object correspondence (Atsma et al., 2016), integration of pre- and postsaccadic information (Ganmor et al., 2015), and object-location binding (Chiu & Golomb, 2025; Lu & Golomb, 2024)—are sensitive or impervious to cognitive control.

At the broader scale, the systematic feature-binding errors occurring early after saccade completion represent one of many cases in which visual perception is altered when spatial attention is in a more dynamic state. The current findings of motivation reducing postsaccadic mixing but not swapping errors in turn raise interesting open questions about top-down cognitive control in other dynamic attentional scenarios, such as when attention is captured, shifted, or divided between locations (Chen et al., 2019; Dowd & Golomb, 2019), objects (Lee & Shomstein, 2013; Shomstein & Johnson, 2013), or items in memory (Brissenden et al., 2023; Scotti et al., 2021).

Finally, it is worth noting that the explicit, blocked reward prospect manipulation implemented in the current study was devised to induce maximal difference in participants’ sustained motivation across conditions, with the goal of asking whether postsaccadic feature interference could be mitigated when participants were trying harder on the task. Building on this work, an interesting follow-up would be to introduce punishments for large misreport (or specifically retinotopic swap) errors to examine whether punishments modulate (or selectively modulate) the retinotopic swap errors. Furthermore, previous research has also found alternative reward effects, including transient reward reinforcement effects occurring on a trial-by-trial basis (Schumann et al., 2022) and learning mechanisms where reward associations are acquired through past experiences (Anderson et al., 2011; Gong & Li, 2014; Infanti et al., 2015; Stankevich & Geng, 2014). Future investigations implementing more nuanced experimental designs (e.g., Steinborn et al., 2017; Wolf et al., 2025) could further dissect the influences of cognitive control, motivation, and reward on perisaccadic feature perception.

In conclusion, the current study investigated how top-down motivation influences postsaccadic feature perception, revealing a multifaceted pattern of postsaccadic feature-binding errors across levels of motivation. These findings suggest roles of reward/motivation in distinct attentional mechanisms underlying postsaccadic remapping and the maintenance of perceptual stability across saccades, altogether highlighting the influences of top-down cognitive control on spatial attention and visual perception in dynamic contexts.

## Supplementary Information

Below is the link to the electronic supplementary material.

Supplementary file1 (PDF 338 kb)

## Footnotes

1 One participant completed only four low-reward blocks and five high-reward blocks due to scheduling conflict, but the number of completed trials remained sufficient for modeling (minimum of 80 trials per condition), so we included their data, per the preregistration.

## Declarations

### Open practices statement

The research background, hypotheses, methods, and analysis plan were preregistered prior to data collection (https://osf.io/amsjb/?view_only=5c323ff94c944b0ba9a067bfa3464278). All study materials, data, and analytic code are publicly available (https://osf.io/djfru/?view_only=1a84eaa8ea1d4f0ca504f25f5f21c653).
