# Supplementary material for: Top-down motivation both decreases and increases feature interference following a saccade
Source: Psychon Bull Rev. 2026 Apr 15;33(4):142. doi: 10.3758/s13423-026-02907-6 (PMC13083521; doi:10.3758/s13423-026-02907-6)
Supplement: Supplementary file 1 — Supplementary file1 (PDF 338 kb) [file 13423_2026_2907_MOESM1_ESM.pdf]

Supplementary Analyses

Exploratory Analyses on Unmodeled Measures of General Performance

In addition to the pre-registered analyses on general performance indicators derived from probabilistic mixture models (i.e., guess rate, standard deviation of the primary target distribution), we performed exploratory analyses on unmodeled measures on general performance (i.e., mean absolute error, standard deviation of absolute response errors; Figure S1). We performed two-way (reward x delay) repeated-measure ANOVA on these measures to examine the effects on reward and post-saccadic delay.

Overall, analysis on the unmodeled measures of general performance yielded similar results to the main results on general performance. Results on mean absolute error showed significant main effects of reward ($F(1, 39) = 6.75$, $p = .013$, $\eta_p^2 = 0.15$, $BF_{incl} = 3.55$) and post-saccadic delay ($F(1, 39) = 66.5$, $p < .001$, $\eta_p^2 = 0.63$, $BF_{incl} = 2.02 \times 10^7$). Participants made smaller response errors when task reward was higher or when the stimulus array appeared later after saccade completion, compared to the low-reward condition or the short post-saccadic delay condition, respectively. The two-way interaction between reward and post-saccadic delay was not significant ($F(1, 39) = 2.97$, $p = .093$, $\eta_p^2 = 0.07$, $BF_{incl} = 0.73$). Results on the standard deviation of absolute response errors were similar. There were significant main effects of reward ($F(1, 39) = 7.14$, $p = .011$, $\eta_p^2 = 0.15$, $BF_{incl} = 2.48$) and post-saccadic delay ($F(1, 39) = 86.73$, $p < .001$, $\eta_p^2 = 0.69$, $BF_{incl} = 3.05 \times 10^8$), suggesting that the response errors were more concentrated when reward was increased or when the delay after saccade completion was longer. The two-way interaction was not significant ($F(1, 39) = 4.01$, $p = .052$, $\eta_p^2 = 0.09$, $BF_{incl} = 1.8$).

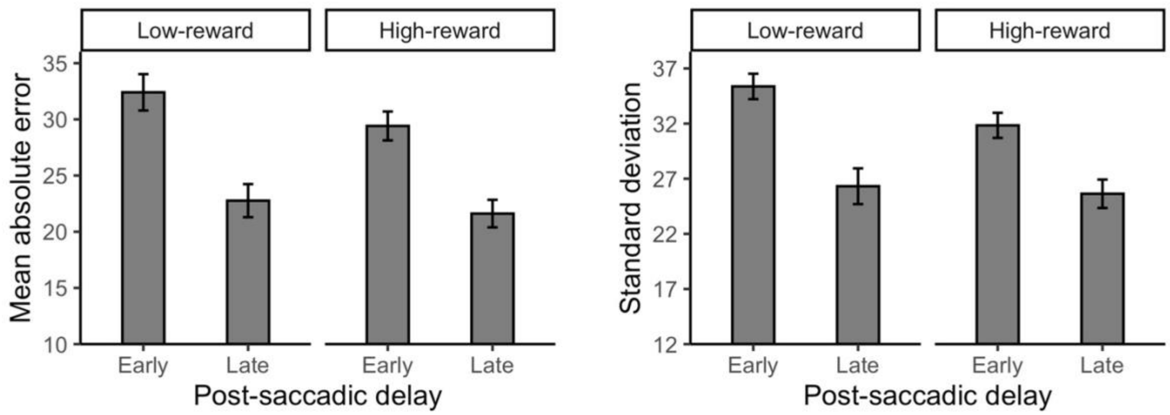

Figure S1. Mean values of unmodeled measures of general performance. Left plot depicts results on mean absolute response error. Right plot depicts results on the standard deviation of absolute response error. Error bars represent 95% confidence interval calculated based on within-participant standard error of the mean (Cousineau, 2005).

Control Analyses to Address Tradeoffs between Mixing and Swapping Errors

In the current results, we found distinct reward modulations on systematic feature-binding errors early after saccade completion. More specifically, there were mixing but not retinotopic swapping errors in the low-reward condition, and retinotopic swapping but not mixing errors in the high-reward condition. However, the mixing and retinotopic swapping errors both concern response errors in the positive direction and could potentially suffer from a tradeoff between the two parameters (μ, β_1) during model fitting. In other words, the distinct patterns of feature-binding errors found across reward conditions might instead reflect interactions between the two parameters during the model fitting process, rather than the underlying attentional mechanisms. We address this alternative explanation through two control analyses.

Correlation between Mean Shift and Retinotopic Misreport Rate

Firstly, we tested the presence of tradeoff between mixing and swapping errors by assessing the relationship between the mean shift parameter (μ) and the retinotopic misreport rate (β_1) parameter. If there was a tradeoff between the two parameters, we should see a negative correlation between the

two parameters. As shown in Figure S1, the relationship between estimates of the mean shift parameter and the retinotopic misreport rate were in the positive direction for low- and high-reward conditions, though the associations were not statistically significant in either condition (Low-reward: $r(38) = 0.22$, $p = .173$; High-reward: $r(38) = 0.24$, $p = .141$). The lack of negative associations between the parameters thus argues against tradeoff between the two parameters as the explanation for the observed results.

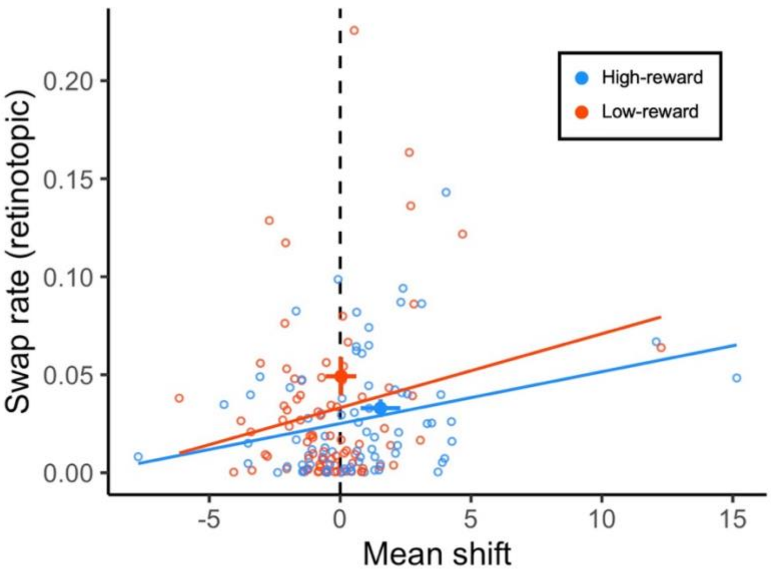

Figure S2. Scatter plot of maximum likelihood estimates of the mean shift (μ) and retinotopic swap rate (β_1) parameters at the early post-saccadic timepoint for both reward conditions. Individual participants are depicted by the unfilled circles. Condition means are depicted by filled circles. Error bars represent 95% confidence interval calculated based on within-participant standard error of the mean (Cousineau, 2005).

Alternative Model without Flexible Mean

In addition, we tested whether the observed variation in retinotopic swapping errors was influenced by the mean shift parameter, by fitting the data to an alternative model without the flexible mean shift parameter (see Eq 1 below). Results showed similar patterns on retinotopic swapping errors, manifested by participants more likely to misreport the retinotopic non-target color compared to the control non-target color only in the high-reward early post-saccadic delay condition ($t(39) = 3.51$, $p = .001$, $d = 0.55$, $BF_{10} = 26.89$) and not in the three remaining conditions ($ps > .14$, $BFs_{10} < 0.49$). These

results suggest that early retinotopic swapping errors occur in the high-reward condition but not in the low-reward condition, regardless of the presence of a flexible mean shift parameter in the model fitting procedure.

$$p(\theta) = (1 - \beta_1 - \beta_2 - \gamma)\phi_{0,\kappa} + \beta_1\phi_{+90°,\kappa} + \beta_2\phi_{-90°,\kappa} + \gamma(\frac{1}{360°}) \quad (1)$$

Taken together, the primary analyses (presented in the main article) showed disparate patterns of systematic feature-binding errors across reward conditions, indicating top-down reward differentially modulates post-saccadic remapping and feature perception. In the two analyses reported here, we found no direct evidence for parameter tradeoff from the estimated values, and the results on swapping errors were comparable with and without the mean shift parameter in the model. These findings demonstrated that the distinct reward modulations on feature-binding errors were not confounded by tradeoff between parameters reflecting the two types of errors, further augmenting the primary results.

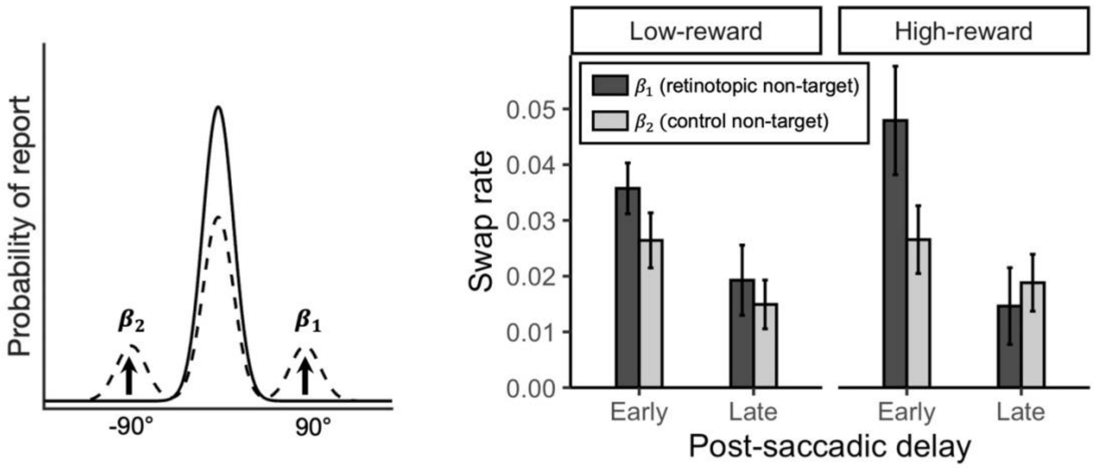

Figure S3 Cartoon illustration for swapping errors (left) and mean maximum likelihood estimates for swap rates based on an alternative model without the flexible mean shift parameter. Error bars represent 95% confidence interval calculated based on within-participant standard error of the mean (Cousineau, 2005).
